# Polycystic kidney disease: The cilia mechanosensation debate gets (bio)physical

**DOI:** 10.1038/s41581-023-00701-4

**Published:** 2023-05-01

**Authors:** Dagmar Wachten, Pleasantine Mill

**Affiliations:** 1 Institute of Innate Immunity, Biophysical Imaging, Medical Faculty, University of Bonn, Bonn, Germany; 2 MRC Human Genetics Unit, Institute for Genetics and Cancer, University of Edinburgh, Edinburgh, UK

## Abstract

For the first time, two new studies applied a mechanical stimulus directly to a cilium, independent of a chemical signal, and demonstrated that force-based bending of a single nodal axoneme is sufficient to induce intraciliary Ca^2+^ flux in a PKD2-dependent manner, which propagated to drive asymmetric gene expression.

Cilia project as microtubule-based protrusions from the surface of most cells and mutations in genes regulating cilia structure and function result in syndromic disorders termed ciliopathies. Cilia are enriched in receptors, channels and effectors, which enables to function as cellular antennae. With diverse roles in chemosensation and, more controversially, in mechanosensation, cilia convert extracellular information — chemical or mechanical stimuli — into an intracellular signal that the cell can interpret. Connecting the flow of this information across space and time remains a challenge *in vivo* as signals traverse from the confined spaces within the cilium to the whole cell and through other cells in the tissue to instruct developmental or homeostatic decisions. Two recent tour-de-force papers by Katoh et al.^1^ and Djenoune et al.^2^ in *Science* elegantly combine cutting-edge light-sheet microscopy, optical tweezers, and biosensors in both mouse and zebrafish embryos to directly tackle this challenge and address one of the most controversial questions in the field: how do cilia instruct left–right (L–R) asymmetry during embryonic development and is mechanosensation by cilia key to this process?

At the heart of the matter is a small, transient, and pit-like embryonic structure called the left–right organizer (LRO; also termed node in mammals). Within the central pit, motile cilia generate a leftward laminar flow, which initiates a signaling cascade that is confined to, and hence specifying, the left side of the embryo. Although the architecture of the node evolves from flat to concave, and the motile cilia position from central to posteriorly tilted, the window for nodal flow-induced signaling is limited to only 6 hours in mice^3^. At the receiving end, the key players in the debate are the sensory (that is, non-motile) cilia that are localized in the crown of the node. Defects in cilia motility and defects in ciliary-based signaling due to abnormalities in the PKD2 ion channel, which localizes to sensory cilia, abolishes symmetry breaking ^4,5^. This cilia-based ‘surveillance system’ is exquisitely sensitive; genetically restoring cilia motility in one or two cilia in the node of mutants with immotile cilia is sufficient to restore L-R patterning^5^. What divides the field in this ‘two cilia model’ of establishing L–R asymmetry is whether chemosensory^6^ or mechanosensory^4,5^ cues are being sensed in the nodal flow. In both models, an asymmetric, left–sided Ca^2+^ gradient is established to trigger a signaling cascade responsible for L-R determination and establishment of the correct biased position, or *situs*, of many visceral organs^4^. But how is the directional flow sensed by cilia? On what scale is the sensing required to trigger the cellular, tissue-level, and organism-level events needed to establish the L–R axes?

Genetic studies have suggested that mechanosensation of sensory cilia underlies sensing of nodal flow, but experiments using microfluidics in combination with Ca^2+^ sensors targeted to cilia or the cytoplasm, failed to find evidence of mechanosensation as cilia did not act as Ca^2+^-responsive mechanosensors^7^. In these landmark papers by Katoh et al.^1^ and Djeunoune et al.^2^, the spatial specificity and tuneable force generation of optical tweezers is harnessed to directly apply mechanical force and ‘bend’ single sensory cilia, mimicking the force of nodal flow in both mouse and zebrafish LRO. Using immotile cilia mutants, cilia Ca^2+^sensors, and markers of L-R symmetry breaking, both studies demonstrate that direct bending of a single cilium is sufficient to induce intraciliary Ca^2+^ transients, which spread to the cell body and neighboring cells of the LRO (Fig.1a,b). Bending was also sufficient to induce asymmetric *Dand5* expression and even reversed defects in cardiac looping, which relies on sensing directional nodal flow. Importantly, bending sensory cilia on the right side of the LRO in mutants with immotile cilia was sufficient to reverse these processes to the right side, leading to *situs inversus*. These responses were lost in loss-of-function *Pkd2* mutants, strongly suggesting that PKD2 is required for transducing direct bending of the cilium into symmetry breaking.

But how is differential cilia bending in response to flow sensed? Katoh and colleagues suggest that the directional flow is converted into strain on the axoneme, which only activates ciliary signaling on the left side of the node owing to the asymmetric localization of PKD2 along the cilia^1^ (Fig.1b). Importantly, this system would require high sensitivity. Similar to a ‘counting and integrating a signal’ model, Djenoune et al suggest that this sensitivity is achieved through a requirement for repetitive and consistent mechanical stimulation of a single cilium before a Ca^2+^ signal will be transmitted^2^. Such a mechanism would discern random from true (repetitive) stimuli, and the threshold for noise buffering is tunable according to the stimulation it encounters.

In these new studies, the researchers have used exceptional techniques to apply, for the first time, a mechanical stimulus directly to a cilium, independent of a chemical signal, and their nanoscale to whole-organism analyses, provide great insight into the establishment of L–R asymmetry. However, not all questions have been answered (Fig.1c). First, neither study can absolutely rule out that a chemical signal exists in parallel to the mechanical stimulation. Second, the Ca^2+^ transients evoked upon bending occur after several seconds, whereas mechanically-activated ion currents (for example, those conduced by Piezo channels) generally occur within milliseconds^8^. This discrepancy raises the question of whether another unknown ‘second messenger’ acts upstream to trigger the Ca^2+^ response. Although the authors have tried to rule out back-propagation of Ca^2+^ from the cytoplasm^7^, the time scales are difficult to reconcile. Third, the applied force necessary to elicit ciliary Ca^2+^ transients on bending are ~0.1 mN/m, which is below the ~3-10 mN/m detection threshold of known, tension-gated ion channels^9^. Thus, the debate of PKD2 being mechanosensitive continues. Last but not least, asymmetric localization of ion channels in motile cilia such as sperm flagella^10^, has been proposed to control rotational movement — could asymmetric PKD2 localization be necessary for a deflection-dependent mechanoresponse? And how is this asymmetry established? Does it evolve by nodal flow?

With another preprint about the chemosensory model^11^, demonstrating leftward transfer of the putative polycystin complex partner for PKD2 — polycystic kidney disease protein 1-like 1 (PKD1L1) — in the node to mediate Ca^2+^ signaling, the final bell in this round of the ciliary mechanosensation debate may not yet be rung.

## Figures and Tables

**Figure 1:**
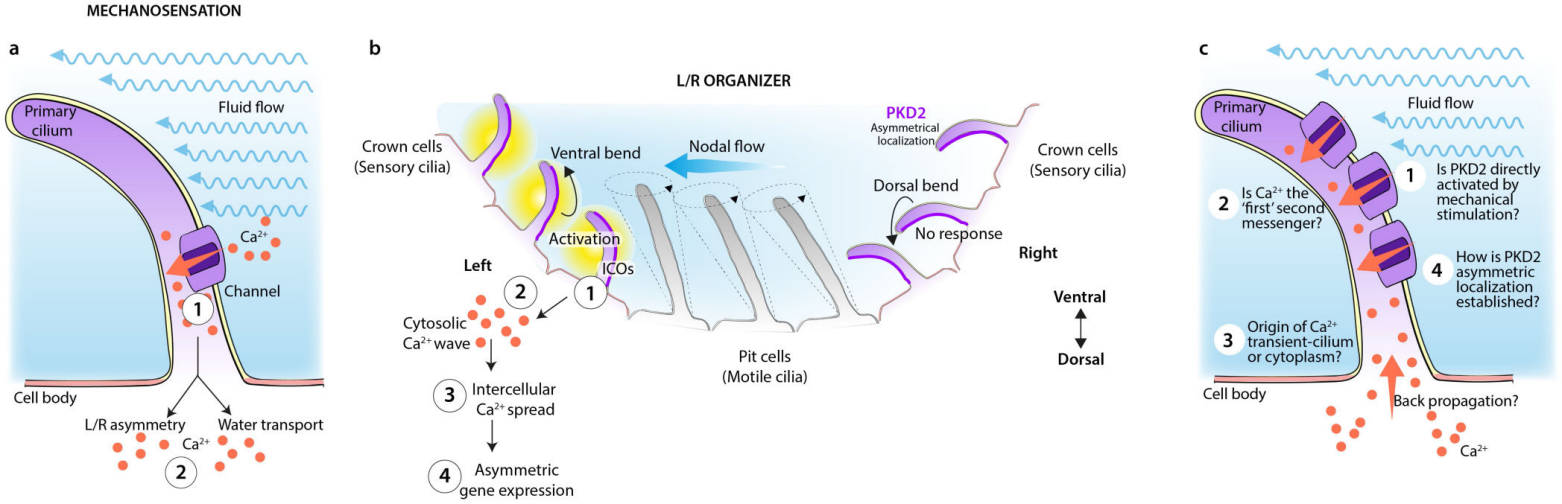
Cilia as mechanosensors. **a,** Strong genetic and cell biological evidence exists for primary cilia to function in mechanotransduction in the embryonic node in response to nodal flow, and in kidney tubule epithelial cells in response to glomerular flow shear stress. Fluid flow leads to opening of Ca^2+^ ion channels, triggering intraciliary Ca^2+^ transients (1), which initiate intracellular signaling (2), leading to asymmetric gene expression or water resorption, respectively. However, one study^7^ concluded that cilia did not function as Ca^2+^-dependent mechanotranducers and that bending under even supraphysiological flow rates was insufficient to trigger intraciliary Ca^2+^ transients. **b,** Schematic of the mouse left-right organizer LRO (also known as node) showing crown cells with sensory cilia and pit cells with motile cilia, which generate the leftward nodal flow. Bending of cilia in response to nodal flow or force applied directly by optical tweezers is sufficient to trigger intraciliary Ca^2+^ transients (ICOs) (1), promoting intracellular Ca^2+^ signaling (2), which eventually spreads to neighboring cells in the LRO and left mesendoderm (3) to result in asymmetric gene expression^1,2^. This whole program could be initiated after bending a single cilium in immotile cilia mutants and was abolished in loss-of-function *Pkd2* mutants. Asymmetric localization of PKD2 (also known as polycystin 2) to clusters enriched along the dorsal side of cilia is proposed to induce a Ca^2+^ response only on the left side of the embryo. **c,** Open questions remain: (1) Given the forces that are necessary to open mechanosensitive ion channels, is PKD2 directly activated by flow-induced mechanical forces?; (2) Given the lag phase between bending and intraciliary Ca^2+^ transients, is Ca^2+^ the true ‘first’ second messenger or is there another messenger acting upstream, possibly initiating an intracellular Ca^2+^ transient that back-propagates^7^ (3)?; (4) How is PKD2 asymmetric localization to the dorsal side of cilia established?
